# Supplementary material for: Intraocular liver spheroids for non-invasive high-resolution in vivo monitoring of liver cell function
Source: Nat Commun. 2024 Jan 26;15:767. doi: 10.1038/s41467-024-45122-4 (PMC10817975; doi:10.1038/s41467-024-45122-4)
Supplement: Supplementary file 1 — Supplementary Information [file 41467_2024_45122_MOESM1_ESM.pdf]

Supplementary Fig. 1

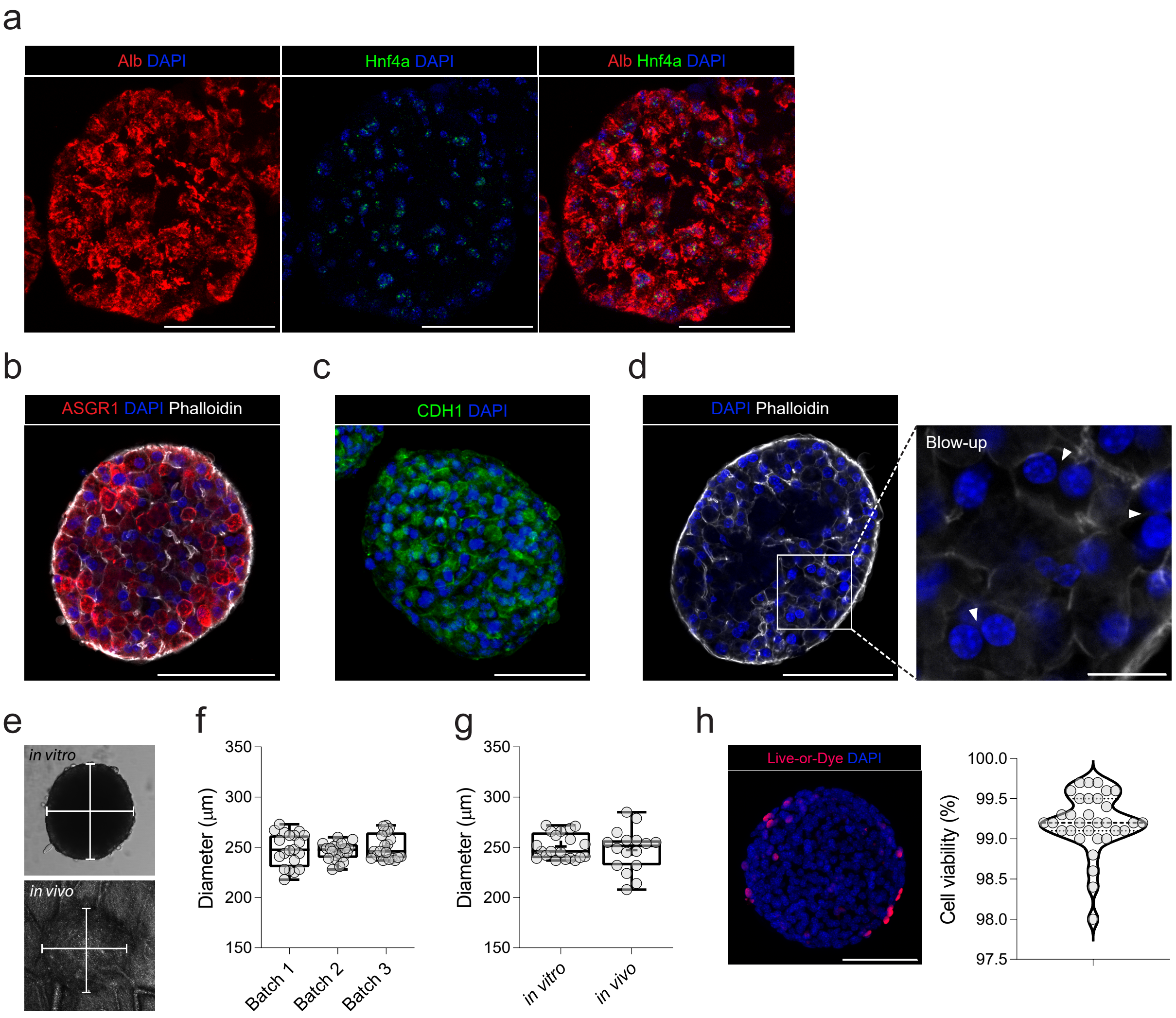

Characterization of liver spheroids prior and after transplantation. a, RNA in situ hybridization showing Alb expression (red), Hnf4α expression (green), and DAPI staining (blue) in liver spheroids in vitro, prior to transplantation. **b,c,d,** Immunofluorescence staining of hepatic marker ASGR1 (red), structural proteins CDH1 (green) and F-actin (Phalloidin, white) and bi-nucleate hepatocytes (white arrowheads) in cultured liver spheroids. **e,** Schematics of spheroid size measurement; an average of vertical and horizontal diameters was calculated. **f,** Size of liver spheroids in vitro, n=3 spheroid batches. Whiskers represent min/max values, with mean shown as '+'; statistical analysis: One-way ANOVA. **g,** Size comparison between liver spheroids prior to transplantation and at 1 month post-tx. Whiskers represent min/max values, with mean shown as '+'; statistical analysis: T-test. **h,** Representative staining of dead cells within a spheroid in vitro by Live-or-Dye (red). Violin plot representing the quantification of cell viability of a representative batch of liver spheroids (1200 cells/well), prior to transplantation. **a,b,c,d,h** scale bars= 100 µm. Source data are provided as a Source Data file.

Supplementary Fig. 2

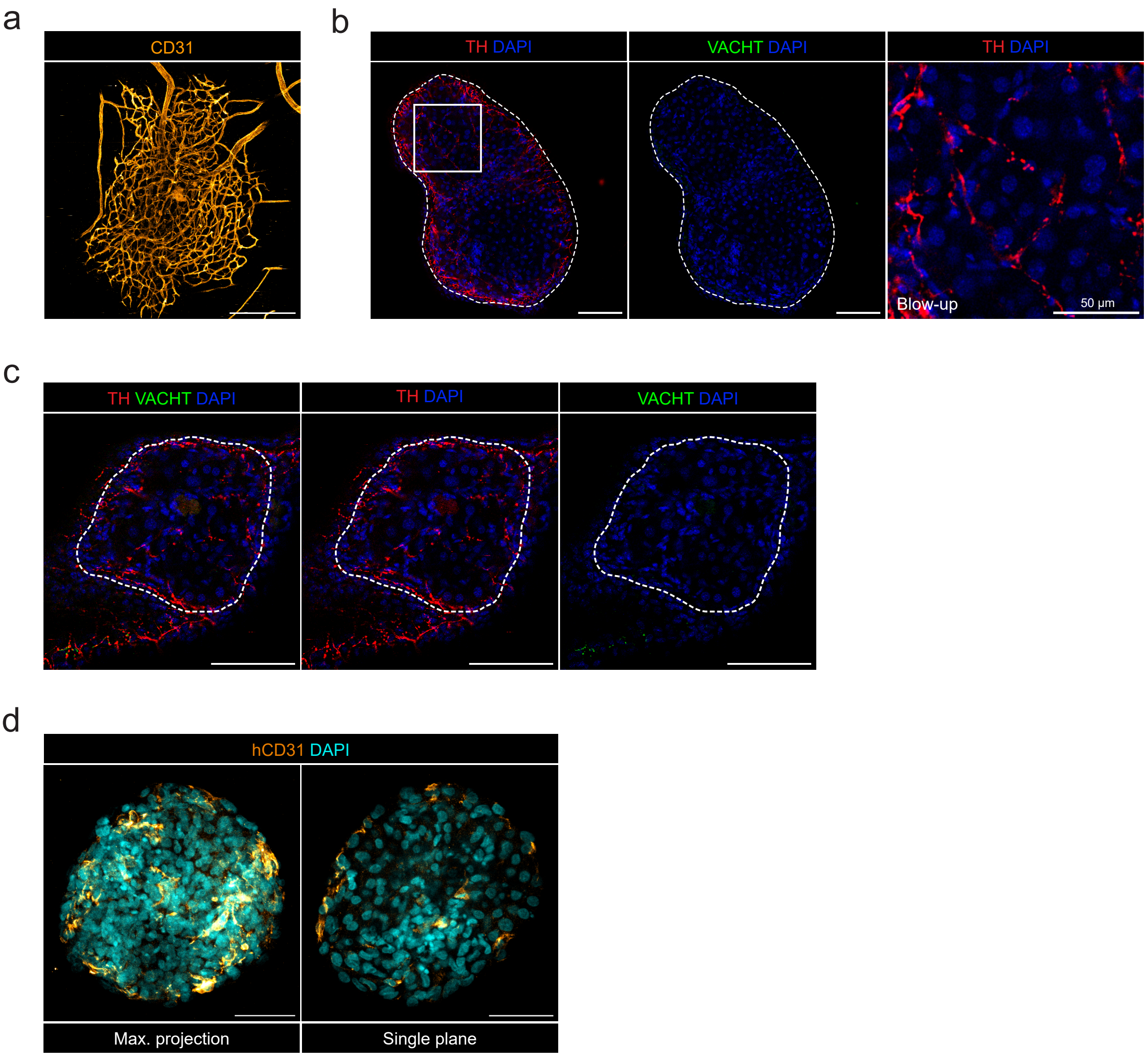

Vascularization and innervation of liver spheroids in the ACE. a, Immunofluorescence staining of intra-spheroid vasculature network (CD31, orange) at 6 months post-tx. Max. projection image. **b,** Immunofluorescence staining of ACE-liver spheroids and surrounding iris tissue, showing intra-spheroid sympathetic (TH, red) and parasympathetic (VACHT, green) nerves at 2 months post-tx. Blow-up showing TH-positive nerves among hepatocytes. Single plane images. **c,** Immunofluorescence staining of intra-spheroid sympathetic (TH, red) and parasympathetic (VACHT, green) innervation at 6 months post-tx. Single plane images. **d,** Immunofluorescence staining of PHH+LSECs human liver spheroids in vitro, prior to transplantation, showing LSECs positive for human-CD31 (hCD31, orange). **a,b,c,d** scale bars = 100 μm.

Supplementary Fig. 3

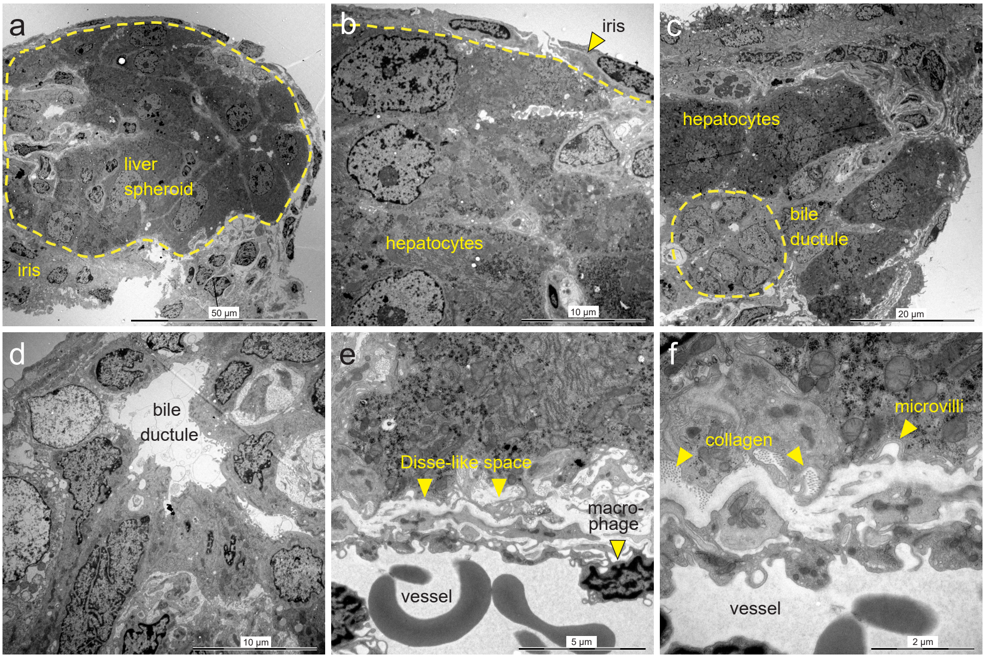

Microscopic structure of the engrafted liver spheroids. TEM images showing tissue architecture and features of ACE-liver spheroids. The liver spheroids become covered by a monolayer of iris cells and the hepatocytes are identified by their large polyhedral shape and dark cytoplasms **(a,b)**; Cholangiocyte-like cells can be seen forming bile ductules within the spheroid mass **(c,d)**; a Disse-like space containing microvilli and collagen fibers is created between hepatocytes and endothelial cells, and macrophages and red blood cells are seen within the vessel lumen **(e,f)**.

Supplementary Fig. 4

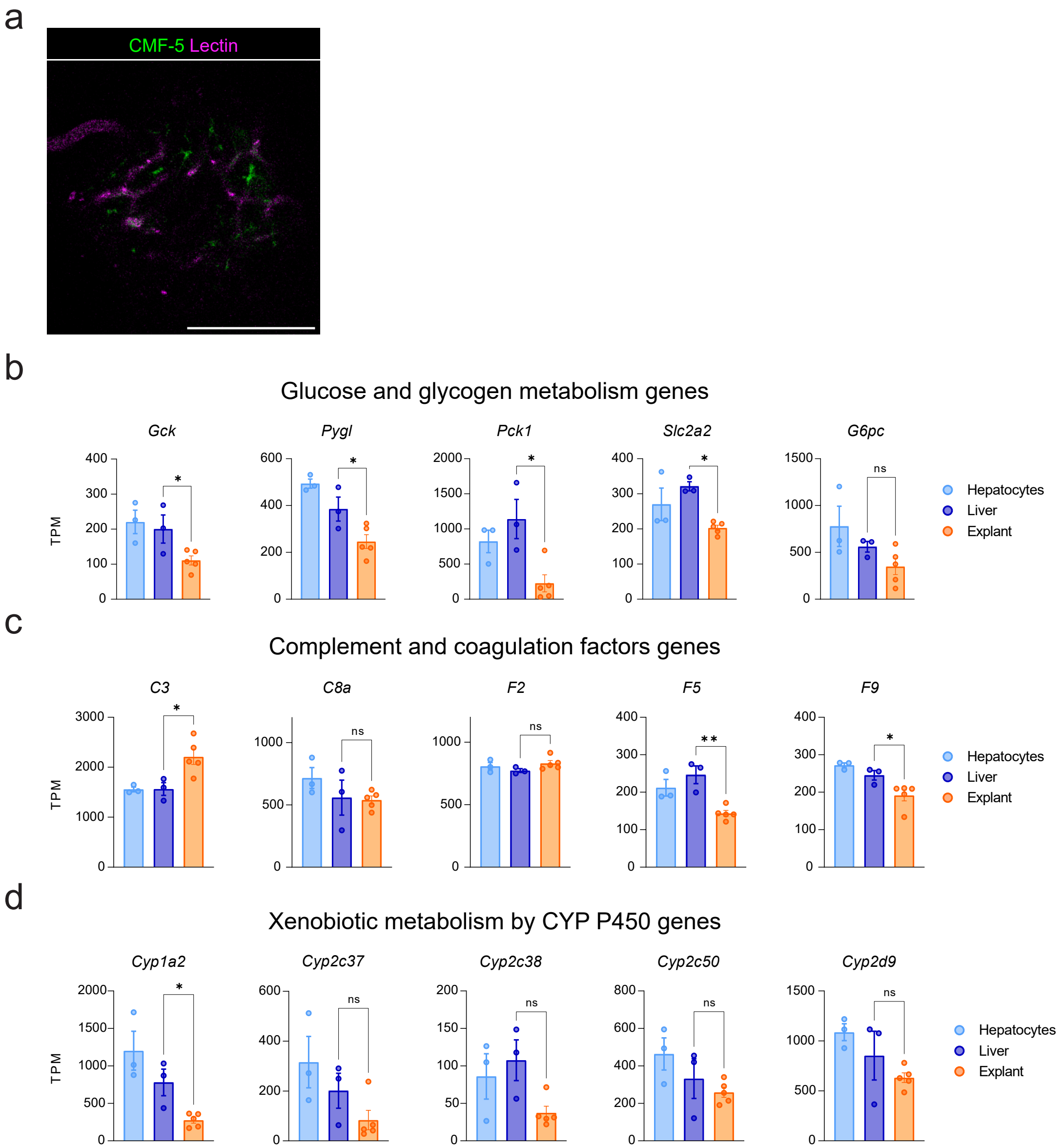

Bile canaliculi network and gene expression of specific hepatocyte functions in engrafted liver spheroids. a, In vivo imaging of bile canaliculi (CMF-5, green) and vessels (lectin, magenta) within ACE-liver spheroids at 6 months post-tx, single plane image, scale bar= 100 µm. **b,** Expression levels of selected glucose and glycogen metabolism genes in explant samples compared to livers at 2 months post-tx. Results shown as TPM, error bars indicate mean ± SEM, *p<0.05 by T-test in all genes, except for Pck1 and Slc2a2, which were analyzed by Mann-Whitney test. **c,** Expression levels of selected complement and coagulation factor genes in explant samples compared to livers at 2 months post-tx. Results shown as TPM, error bars indicate mean ± SEM, *p<0.05 by T-test in all genes, except for F9, which was analyzed by Mann-Whitney test. **d,** Expression levels of selected xenobiotic metabolism genes in explant samples compared to livers at 2 months post-tx. Results shown as TPM, error bars indicate mean ± SEM, *p<0.05 by T-test in all genes, except for Cyp2c37 and Cyp2c38, which were analyzed by Mann-Whitney test, b,c,d n= 3 freshly isolated hepatocytes, 3 livers, 5 explants. Source data are provided as a Source Data file.

Supplementary Fig. 5

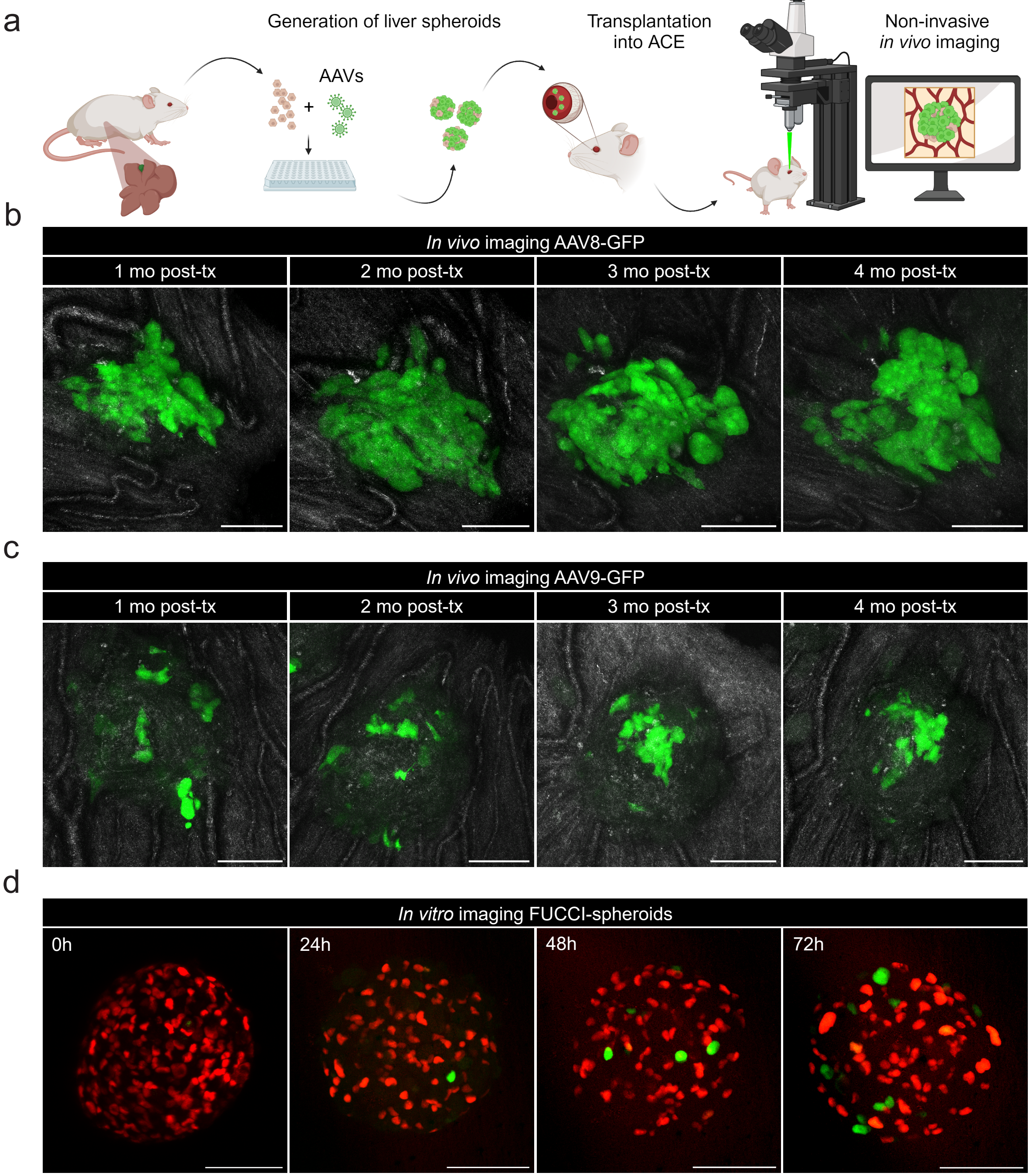

Expression of fluorescent probes in liver spheroids for transplantation. a, Experimental design: primary liver cells enriched in hepatocytes were incubated in vitro with adeno-associated viruses (AAVs) during spheroid formation. Spheroids were transplanted into the ACE of recipient mice, where they can be imaged in vivo longitudinally. **b,** In vivo imaging at monthly intervals post-tx of ACE-liver spheroids transduced with AAV8 expressing GFP (green) under the CAG promoter. **c,** In vivo imaging at monthly intervals post-tx of ACE-liver spheroids transduced with AAV9 expressing GFP (green) under the CAG promoter. **d,** In vitro imaging over 72h of FUCCI-expressing liver spheroids treated with YAC proliferation cocktail. b,c,d maximum projection images, scale bar= 100 μm.

Supplementary Fig. 6

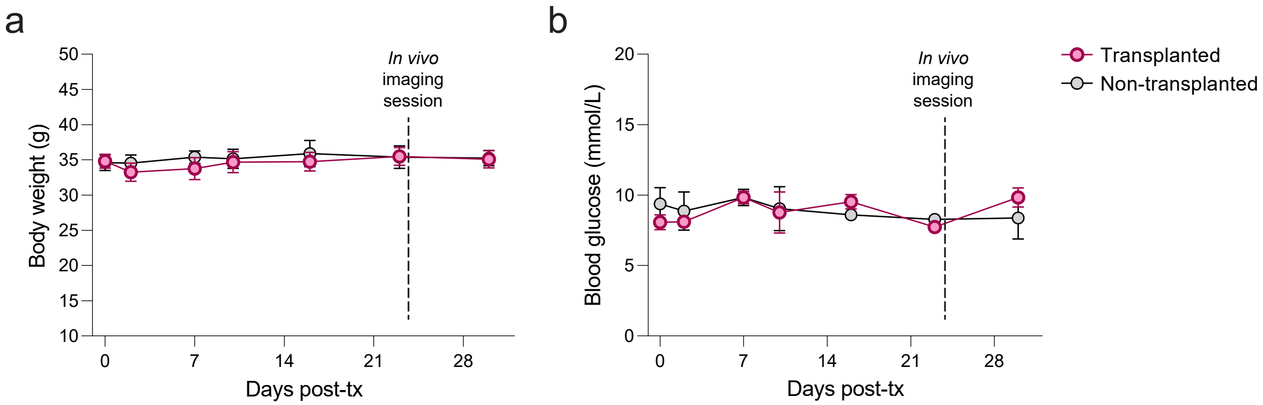

Metabolic features of transplanted and non-transplanted animals. a, Body weight (progression) and **b,** blood glucose levels in transplanted and non-transplanted mice over 1-month post-tx. One in vivo imaging session was performed on day 24 only in transplanted mice. N=3 mice per group. Statistical analysis: Two-way ANOVA. Source data are provided as a Source Data file.

Supplementary Table 1. Medical and demographic information of Primary Human Hepatocytes (PHH) and primary Liver Sinusoidal Endothelial Cell (LSEC) donors.

Donor	Cell type	Sex	Ethnicity	Age	Cause of death	Relevant medical and social history
1	PHH	F	Hispanic	30	Head trauma	Smoker, narcotic dependency
2	PHH	F	Caucasian	42	Anoxia due to drug intoxication	Drug abuse, substance abuse, mood disorder, smoker
3	LSEC	F	Hispanic	52	No information available	No information available

Supplementary Table 2. Confocal Leica SP5 microscope settings used for intraocular *in vivo* imaging of liver spheroids. LPm: laser power from manufacturer; Ex: λ excitation; Em: λ emission; L int: laser intensity; Po: optical power at the sample plane measured by using Thorlabs PM100USB Power and Energy Meter Interface with USB Operation combined with Thorlabs S170C Microscope Slide Photodiode Power Meter Sensor (Thorlabs Inc. Newton, New Jersey, U.S.); Pi: pinhole; Z: stack step size.

**Probe/ protein**	**LPm (mW)**	**L int (%)**	**Ex (nm)**	**Em (nm)**	**Po (µW)**	**Pi (AU)**	**Format (pixels)**	**Speed (Hz)**	**Z (µm)**	**Acquisition time**
Lectin DyLight-649	~10	20	633	650-700	~68.0	1	512x512	600	4	~1 min / spheroid
Lectin FITC	~20	20	488	510-540	~7.0	8.4	256x256	600	n/a	Video: ~1 min/spheroid
DiD dye (RBC labelling)	~10	15	633	650-700	~40.0	8.4	256x256	600	n/a	Video: ~1 min/spheroid
Green CMFDA	~20	15	488	510-540	~4.3	1	512x512	600	4	Video or stacks: ~1 min / spheroid
pHrodo Red-LDL	~20	15	561	570-620	~23.8	1	512x512	600	4	~1 min / spheroid
SF44	~20	20	488	580-700	~7.0	1	512x512	600	4	~1 min / spheroid
AAV8/9-GFP	~20	15	488	490-540	~4.3	1	512x512	600	4	~1 min / spheroid
FUCCI	~20	20	488	mAG: 490-530 / mKO2: 560-580	~7.0	1	512x512	600	4	~1 min / spheroid

Supplementary Table 3. Antibodies used for immunofluorescence staining

**Antibody**	**Catalog number**	**Vendor**	**RRID**	**Dilution**
α-ASGR1	11739-1-AP	Proteintech, Illinois, US	AB_2059675	1:100
α-CD31	AF3628	R&D Systems, Minnesota, US	AB_2161028	1:200
Human α-CD31 (hCD31)	BBA7	R&D Systems	AB_356960	1:100
α-TH	213104	Synaptic Systems, Göttingen, Germany	AB_2619897	1:100
α-VACHT	139103	Synaptic Systems	AB_887864	1:100
α-F4/80	MCA497RT	BioRad, California, US	AB_1102558	1:100
α-GLUT2	NBP2-22218	Novus Biologicals, Colorado, US	AB_2335858	1:100
α-CDH1	3195	Cell Signalling, Massachusetts, US	AB_2291471	1:100
α-KI67	ab15580	Abcam, Cambridge, UK	AB_443209	1:200
