# Supplementary material for: Intraocular liver spheroids for non-invasive high-resolution in vivo monitoring of liver cell function
Source: Nat Commun. 2024 Jan 26;15:767. doi: 10.1038/s41467-024-45122-4 (PMC10817975; doi:10.1038/s41467-024-45122-4)
Supplement: Supplementary file 3 — Description of Additional Supplementary Files [file 41467_2024_45122_MOESM3_ESM.pdf]

Description of Additional Supplementary Files

Supplementary Movie 1. In vivo imaging of labeled red blood cells (red) through intra-spheroid vessels (lectin, white).

Supplementary Movie 2. CMF-5 (green) fluorescent signal collected during in vivo imaging moving the z-plane through the spheroid engrafted in the ACE.
