# Supplementary material for: Intraocular liver spheroids for non-invasive high-resolution in vivo monitoring of liver cell function
Source: Nat Commun. 2024 Jan 26;15:767. doi: 10.1038/s41467-024-45122-4 (PMC10817975; doi:10.1038/s41467-024-45122-4)
Supplement: Supplementary file 6 — Reporting Summary [file 41467_2024_45122_MOESM6_ESM.pdf]

nature portfolio

Corresponding author(s): Noah Moruzzi

Last updated by author(s): Jan 12, 2024

Reporting Summary

Nature Portfolio wishes to improve the reproducibility of the work that we publish. This form provides structure for consistency and transparency in reporting. For further information on Nature Portfolio policies, see our Editorial Policies and the Editorial Policy Checklist.

Statistics

For all statistical analyses, confirm that the following items are present in the figure legend, table legend, main text, or Methods section.

n/a	Confirmed	
☐	☒	The exact sample size (*n*) for each experimental group/condition, given as a discrete number and unit of measurement
☐	☒	A statement on whether measurements were taken from distinct samples or whether the same sample was measured repeatedly
☐	☒	The statistical test(s) used AND whether they are one- or two-sided<br>*Only common tests should be described solely by name; describe more complex techniques in the Methods section.*
☒	☐	A description of all covariates tested
☐	☒	A description of any assumptions or corrections, such as tests of normality and adjustment for multiple comparisons
☐	☒	A full description of the statistical parameters including central tendency (e.g. means) or other basic estimates (e.g. regression coefficient) AND variation (e.g. standard deviation) or associated estimates of uncertainty (e.g. confidence intervals)
☐	☒	For null hypothesis testing, the test statistic (e.g. *F*, *t*, *r*) with confidence intervals, effect sizes, degrees of freedom and *P* value noted<br>*Give P values as exact values whenever suitable.*
☒	☐	For Bayesian analysis, information on the choice of priors and Markov chain Monte Carlo settings
☒	☐	For hierarchical and complex designs, identification of the appropriate level for tests and full reporting of outcomes
☒	☐	Estimates of effect sizes (e.g. Cohen's *d*, Pearson's *r*), indicating how they were calculated

Our web collection on statistics for biologists contains articles on many of the points above.

Software and code

Policy information about availability of computer code

Data collection	HiSeq 3000
Data analysis	TopHat2, UMI-tool dedup, Samtools, featureCounts, DEseq2, R 4.1.2, GraphPad Prism 9.5.1, ImageJ 1.53t

For manuscripts utilizing custom algorithms or software that are central to the research but not yet described in published literature, software must be made available to editors and reviewers. We strongly encourage code deposition in a community repository (e.g. GitHub). See the Nature Portfolio guidelines for submitting code & software for further information.

Data

Policy information about availability of data

All manuscripts must include a data availability statement. This statement should provide the following information, where applicable:

- Accession codes, unique identifiers, or web links for publicly available datasets
- A description of any restrictions on data availability
- For clinical datasets or third party data, please ensure that the statement adheres to our policy

The RNAseq dataset for sequencing analysis generated in this study have been deposited in the GEO database under the accession code GSE245944 (https://www.ncbi.nlm.nih.gov/geo/query/acc.cgi?acc=GSE245944). Source data used to generate the graphs in the figures are provided with this paper. The rest of the data as images are available from the corresponding author upon reasonable request.

Research involving human participants, their data, or biological material

Policy information about studies with human participants or human data. See also policy information about sex, gender (identity/presentation), and sexual orientation and race, ethnicity and racism.

Reporting on sex and gender	n/a
Reporting on race, ethnicity, or other socially relevant groupings	n/a
Population characteristics	n/a
Recruitment	n/a
Ethics oversight	n/a

Note that full information on the approval of the study protocol must also be provided in the manuscript.

Field-specific reporting

Please select the one below that is the best fit for your research. If you are not sure, read the appropriate sections before making your selection.

[x] Life sciences [ ] Behavioural & social sciences [ ] Ecological, evolutionary & environmental sciences

For a reference copy of the document with all sections, see nature.com/documents/nr-reporting-summary-flat.pdf

Life sciences study design

All studies must disclose on these points even when the disclosure is negative.

Sample size	No power analysis for sample size calculation was performed in our work. The least amount of animals for experiments used was n=3 in the RNAseq data and 4 in the dietary experiments in fig.6. For the rest of the experiments we used a higher n number depending on the spheroid transplanted and animal availability. The sample size was kept at the minimum throughout the work to reduce the amount of experimental animal which would be enough to identify biological differences between the groups.
Data exclusions	Each dataset of experimental values was run for outliers identification using GraphPad Prism outliers calculator with ROUT method and Q = 1% before performing statistical analysis. The outliers identified with the software were removed from the dataset.
Replication	Each experiments shown in the manuscript includes at least 3 biological replicates.
Randomization	All the experimental animals were assigned randomly to each experimental group when 2 or more groups were compared.
Blinding	Blinding was not carried out due to the unlikeliness of bias in our experiments.

Reporting for specific materials, systems and methods

We require information from authors about some types of materials, experimental systems and methods used in many studies. Here, indicate whether each material, system or method listed is relevant to your study. If you are not sure if a list item applies to your research, read the appropriate section before selecting a response.

Materials & experimental systems

n/a	Involved in the study	
[ ]	[x]	Antibodies
[x]	[ ]	Eukaryotic cell lines
[x]	[ ]	Palaeontology and archaeology
[ ]	[x]	Animals and other organisms
[x]	[ ]	Clinical data
[x]	[ ]	Dual use research of concern
[x]	[ ]	Plants

Methods

n/a	Involved in the study	
[x]	[ ]	ChIP-seq
[x]	[ ]	Flow cytometry
[x]	[ ]	MRI-based neuroimaging

Antibodies

Antibodies used	Anti-ASGR1, Proteintech 11739-1-AP, RRID: AB_2059675; Anti-CD31, R&D Systems, AF3628, RRID: AB_2161028; Human anti-CD31, R&D Systems, BBA7, RRID: AB_356960; Anti-TH, Synaptic Systems, 213104, RRID: AB_2619897; Anti-VACHT, Synaptic Systems, 139103, RRID: AB_887864; Anti-F4/80, BioRad, MCA497RT, RRID: AB_1102558; Anti-GLUT2, Novus Biologicals, NBP2-22218, RRID:

	AB_2335858; Anti-CHD1, Cell Signalling, 3195, RRID: AB_2291471; Anti-KI67, Abcam, ab15580, RRID: AB_443209
Validation	Anti-ASGR1 (11739-1-AP): validated by manufacturer for IF in rat liver and shows reactivity in mouse liver tissue for WB and IP; Anti-CD31 (AF3628): validated by manufacturer for IHC in mouse endothelioma cell line and mouse embryo; Human anti-CD31 (BBA7): validated by manufacturer for IHC in HUVEC human cells and human artery; Anti-TH (213104): validated by users for IHC in 8 mouse tissues and 1 rat tissue (see manufacturer website for more details); Anti-VACHT (139103): validated by users for IHC in over 80 different studies for mouse and rat tissues (see manufacturer website for more details); Anti-F4/80 (MCA497RT): validated by manufacturer for IF in mouse tissue; Anti-GLUT2 (NBP2-22218): validated by manufacturer for IF and by users in 10 studies in mouse tissues (see manufacturer website for more details); Anti-CDH1 (3195): validated by manufacturer in human breast cancer line and cited in over 15 studies for application in mouse tissues (see manufacturer website for more details); Anti-KI67 (ab15580): validated by manufacturer for IF in human tissue and reviewed by 6 users for IF in mouse tissue and cell lines.

Animals and other research organisms

Policy information about studies involving animals; ARRIVE guidelines recommended for reporting animal research, and Sex and Gender in Research

Laboratory animals	The mice age was between 3-9 months. The strains used were the following: B6(Cg)-Tyrc-2J/J mice (Albino B6/J mice); NOD.Cg-Prkdcscid Il2rgtm1Wjl/SzJ (NSG) mice; B6.Cg-Tg(Fucci)504Bsi Tg(Fucci)596Bsi (FUCCI) mice; C57B6J mice.
Wild animals	The study did not involve wild animals
Reporting on sex	The sex was not considered in the study design beside the dietary experiments in figure 6, where male mice were used due to more dramatic diet induced obesity phenotype. The other findings are applicable for both females and males, therefore this information was not collected and no sex based analysis was performed.
Field-collected samples	The study did not involve field collected samples
Ethics oversight	All animal experiments were performed in accordance with the Animal Experiment Ethics Committee at Karolinska Institutet. This work falls under the broader ethical permission approved by Jordbruksverket: “Studies on the function of hormone-releasing and hormone-stimulated cells and related cells/tissues in normal and diabetic animal models and in transplanted tissues and cells” (n. 6362-2023, previous 16454-2022, 17431-2021, 8822-2020).

Note that full information on the approval of the study protocol must also be provided in the manuscript.
